# *PCAT6* May Be a Whistler and Checkpoint Target for Precision Therapy in Human Cancers

**DOI:** 10.3390/cancers13236101

**Published:** 2021-12-03

**Authors:** Feng Jiang, Qiaoyi Lv, Cexun Hu, Zhanghui Li, Haojie Wu, Shujun Gao, Hui Wang, Yangjing Zhao, Qixiang Shao

**Affiliations:** 1 Jiangsu Key Laboratory of Medical Science and Laboratory Medicine, Department of Immunology, Reproductive Sciences Institute, School of Medicine, Jiangsu University, Zhenjiang 212013, China; jiang_wax@163.com (F.J.); 2212113104@stmail.ujs.edu.cn (Q.L.); 2211913059@stmail.ujs.edu.cn (C.H.); 2212013001@stmail.ujs.edu.cn (Z.L.); whjsnow1990@126.com (H.W.); gaoshujun95@163.com (S.G.); 1000004613@ujs.edu.cn (H.W.); 2 Jiangsu Key Laboratory of Medical Science and Laboratory Medicine, Department of Pathology, School of Medicine, Jiangsu University, Zhenjiang 212013, China; 3 Institute of Medical Genetics and Reproductive Immunity, School of Medical Science and Laboratory Medicine, Jiangsu College of Nursing, Huai’an 223002, China

**Keywords:** lncRNA, *PCAT6*, biological function, molecular mechanism, signaling pathway, oncogenic biomarker

## Abstract

**Simple Summary:**

Prostate cancer-associated transcript 6 (*PCAT6*), as a newly discovered carcinogenic long non-coding RNA (lncRNA), is abnormally expressed in multiple diseases. With the accumulation of studies on *PCAT6*, we have a deeper understanding of its biological functions and mechanisms. Therefore, in this review, the various molecular mechanisms by which *PCAT6* promotes multiple tumorigenesis and progression are summarized and discussed. Furthermore, its potential diagnostic, prognostic, and immunotherapeutic values are also clarified.

**Abstract:**

LncRNAs are involved in the occurrence and progressions of multiple cancers. Emerging evidence has shown that *PCAT6*, a newly discovered carcinogenic lncRNA, is abnormally elevated in various human malignant tumors. Until now, *PCAT6* has been found to sponge various miRNAs to activate the signaling pathways, which further affects tumor cell proliferation, migration, invasion, cycle, apoptosis, radioresistance, and chemoresistance. Moreover, *PCAT6* has been shown to exert biological functions beyond ceRNAs. In this review, we summarize the biological characteristics of *PCAT6* in a variety of human malignancies and describe the biological mechanisms by which *PCAT6* can facilitate tumor progression. Finally, we discuss its diagnostic and prognostic values and clinical applications in various human malignancies.

## 1. Introduction

In recent years, with the development of RNA sequencing (RNAseq) technology, such as next-generation sequencing (NGS) and the third generation sequencing (TGS), more and more non-coding RNAs (ncRNAs) have been discovered, which occupy the majority of the transcriptome (~90%) [1]. Unlike protein-coding genes, ncRNAs do not encode proteins or short peptides. According to whether they are more than 200 nucleotides in length, ncRNAs are divided into two categories: short ncRNAs and long ncRNAs (lncRNAs) [2,3]. LncRNAs participate in a variety of biological processes, such as individual ontogenetic development, cell or tissue development and differentiation, cell proliferation, cell death, cell cycle, migration, and invasion [4,5,6], and numerous pathological conditions, including cancer [7,8,9]. Moreover, lncRNAs play indispensable roles in regulating gene expression via epigenetic modification, transcriptional activation or interference, and post-transcriptional mechanism [10]. For example, lncRNAs can affect biological processes in various cancers by acting as competing endogenous RNAs (ceRNAs) or the sponge of microRNAs [11,12,13], or by directly regulating target proteins [14,15]. Recently, increasing evidence has revealed that lncRNAs are closely related to the clinicopathological features of various diseases, and have better diagnostic and prognostic value [16,17]. In short, lncRNAs are likely to serve as tumor biomarkers and therapeutic targets [18,19].

Prostate cancer-associated transcript 6 (*PCAT6*) is a novel lncRNA that extensively promotes multiple cancer progression. Many investigations have demonstrated that the expression of *PCAT6* is up-regulated in a variety of cancers and closely related to the occurrence and development of tumors [20,21]. Therefore, *PCAT6* may be a potential target for cancer diagnosis and treatment. This review aims to summarize the abnormal expression and subcellular localization of *PCAT6*, and to further understand the underlying mechanisms, and its diagnostic or prognostic values in multiple human cancers.

## 2. The Discovery of *PCAT6*

**Figure 1:**
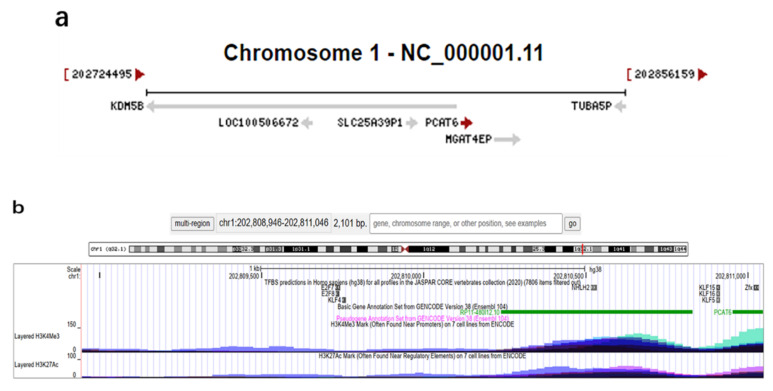
Transcription direction and upstream transcription factors of *PCAT6*. (**a**) The red arrow following *PCAT6* points in the direction of transcription. Reference information comes from the National Center for Biotechnology Information (https://www.ncbi.nlm.nih.gov/gene/100506696 (accessed on 13 July 2021)). (**b**) Transcription factors that may regulate *PCAT6*, including NHLH2. Reference information comes from the University of California Santa Cruz (UCSC) Genome Browser (http://genome.ucsc.edu/ (accessed on 13 July 2021)).

*PCAT6*, a newly discovered lncRNA, is also named *KDM5B-AS1*, *KDM5BAS1*, *PCAN-R1*, *ncRNA-a2*, or *onco-lncRNA-96*. It was first described as *ncRNA-a2* in 2010 [22]. The gene of *PCAT6* is located on chromosome 1q32.1 and contains two exons. It consists of 968 bp and has two transcript variants: transcript variant 1 (NR_046325.1) and transcript variant 2 (NR_046326.1). The transcription direction of *PCAT6* is positive (Figure 1a), and some transcription factors that bind to the promoter region can be found near the upstream 2000 bp (chr1:202808946) and downstream 100 bp (chr1:202811046) of its transcription start sites (chr1:202810946). In addition, the histones H3K4Me3 and H3K27Ac that recognize the promoter sequences are also found in this region, which further proves the existence of an active promoter. Moreover, the transcription factors near these regions are more likely to become the upstream transcription factors of *PCAT6* to regulate its transcription (e.g., nescient helix-loop-helix 2, NHLH2) (Figure 1b). By analyzing the RNAseq data of normal tissue from the Human Protein Atlas database, it was found that human testis tissues had the highest expression level of *PCAT6* [23]. Subsequently, Wan et al. first found that knockdown of *PCAT6* inhibited cell proliferation, invasion, and increased early apoptosis in lung cancer [20]. Increasing numbers of studies have illustrated that high expression levels of *PCAT6* are associated with many human cancers.

## 3. Expression and Subcellular Localization of *PCAT6*

### 3.1. The Abnormal Expression of PCAT6 in Cancers

The expression of *PCAT6* is found to be aberrantly elevated in various human tumor tissues and cell lines compared with matched normal ones, including bladder cancer (BC) [21,24,25], breast cancer (BrCa) [26,27], cervical cancer (CC) [28,29], colorectal cancer (CRC) [30,31], gastrointestinal stromal tumor (GIST) [32], gastric cancer (GC) [33,34], glioblastoma (GBM) [35], hepatocellular carcinoma (HCC) [36,37,38], lung cancer (LC) [20,39,40,41,42,43], osteosarcoma (Osa) [44,45,46], ovarian cancer (OvCa) [47,48], cholangiocarcinoma (CCA) [49], pituitary adenoma (PA) [50], pancreatic ductal adenocarcinoma (PDAC) [51], and prostate cancer (PCa) [52,53]. In subsequent experiments on the biological functions of tumor cells, it has been revealed that a high level of *PCAT6* has strong cancer-promoting effects, mainly including the promotion of cell proliferation, enhancement of migration, invasion and EMT process, as well as the inhibition of cell apoptosis. Meanwhile, *PCAT6* has been shown to promote tumor growth and metastasis in xenograft mouse models (Table 1) [26,29,30,38,39,40,43,44,46,50,52,53,54]. However, Amelia et al. reported that the expression level of *PCAT6* was opposite in lung tumor tissues and lung cancer cell lines compared with the normal control group [55]. Compared with paired normal tissue, *PCAT6* expression level is higher in lung tumors, while its level is lower in non-small cell lung cancer (NSCLC) cell lines compared to the normal human fetal lung fibroblast cell line (IMR-90) [55]. Interestingly, Tu et al. found that, compared to T cells, B cells, dendritics, and neutrophils, *PCAT6* expression was the highest in macrophages which derived from patients of CCA, especially M2 macrophages [54]. Furthermoer, *PCAT6* expression level is also significantly higher in the blood samples of some cancer patients, including BC [24] and LC [41,56]. Contradictorily, Siddique et al., testified that *PCAT6* level had no significant difference in the blood between Saudi CRC patients and healthy donors [57]. It is speculated that the cause of this result may be ethnically related, and the expression level of *PCAT6* in CRC patients of different races might be different.

### 3.2. The Subcellular Localization of PCAT6 in Cancer Cell Lines

LncRNAs play diverse functions depending on different subcellular or extracellular compartmental localizations. Most studies indicate that *PCAT6* is primarily located in the cytoplasm of BC [25], GIST [32], GBM [35], Osa [44,45], PA [50], and Pca [52] cells. Cytoplasmic lncRNAs regulate genes at the translational and post-transcriptional levels, such as interaction with cytoplasmic proteins [58], and interaction with microRNAs to regulate downstream mRNA levels [59,60,61,62,63]. Shi et al. determined that *PCAT6* was principally distributed in the nucleus of NSCLC cells [40]. Nucleic lncRNAs regulate genes at the epigenetic and transcriptional levels, including histone modifications [64,65], DNA methylation [66], and chromatin remodeling [67]. Furthermore, Dong and Lang et al. demonstrated that *PCAT6* was located in both the cytoplasm and nucleus of BrCa and PCa cells by fluorescence in situ hybridization (FISH) and subcellular fraction assays, which was different from most studies [26,53]. This is similar to the lncRNA *HOTAIR*, which regulates genes at both the epigenetic and transcriptional levels, as well as at the post-transcriptional level [68].

As shown in Figure 1a, *PCAT6* is an antisense RNA of *KDM5B*, so it is also known as *KDM5B-AS1*. The RNAs *PCAT6* and *KDM5B* are adjacent in chromosome position. Several antisense lncRNAs have been found to form RNA–RNA dimers with adjacent mRNA, thus improving the stability of mRNA and upregulating mRNA expression levels. For instance, lncRNA *BACE1-AS1* can reduce the degradation of BACE1 by forming a dimer with BACE1 [69]; lncRNA *FGFR3-AS1* promotes Osa through increasing FGFR3 stability [70]. Hence, we speculate that *PCAT6* may also maintain the stability of KDM5B mRNA and upregulate the expression of the KDM5B protein, thus promoting disease progression, but this hypothesis needs to be further confirmed. Furthermore, *PCAT6* is also regulated by different transcription factors. In addition to NHLH2 near H3K4Me3 and H3K27Ac (Figure 1b), transcription factors that have been confirmed to regulate *PCAT6* include the Sp1 transcription factor (SP1) [71] and the enhancer of zeste homolog 2 (EZH2) [30,40].

## 4. *PCAT6* Promotes Cancer Progression by ceRNA Mechanisms

**Figure 2:**
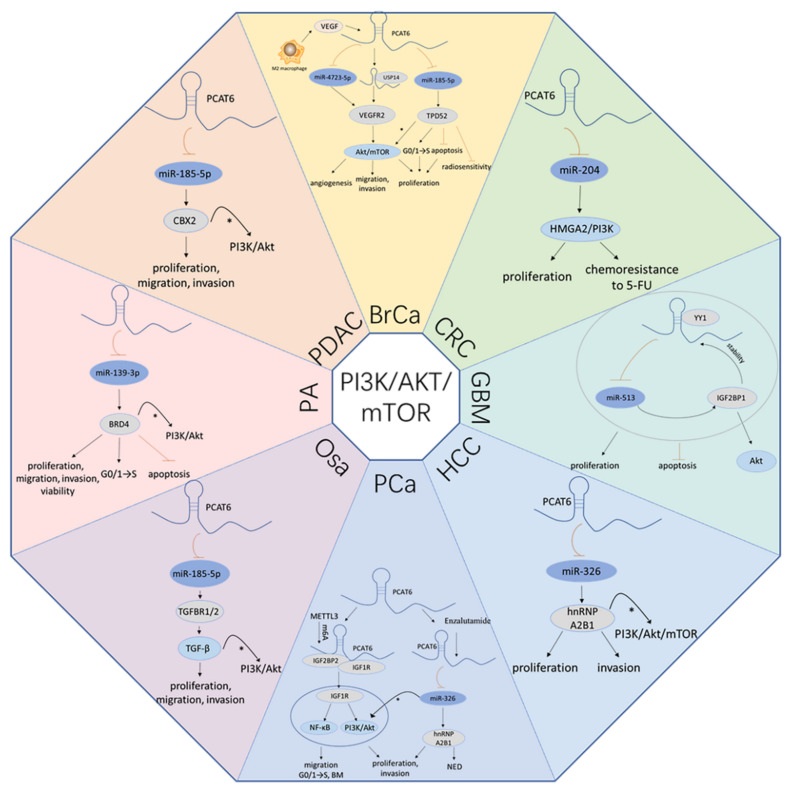
*PCAT6* promotes PI3K/AKT/mTOR signaling Pathway. By sponging miRNAs, *PCAT6* affects the PI3K/AKT/mTOR signaling pathway in multiple, cancers, including BrCa, CRC, GBM, HCC, PCa, Osa, PA, PDAC. *: *PCAT6* is likely to activate the PI3K/AKT/mTOR signaling by regulating ceRNA mechanisms.

**Figure 3:**
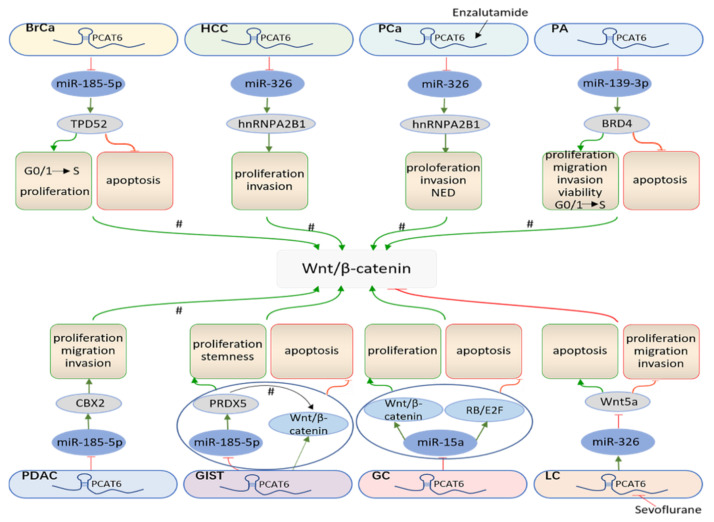
*PCAT6* accelerates the Wnt/β-catenin signaling pathway. By sponging miRNAs, *PCAT6* affects the Wnt/β-catenin signaling pathway in various cancers, including BrCa, HCC, Pca, PA, PDAC, GIST, GC, LC. #: *PCAT6* is likely to activate Wnt/β-catenin signaling by absorbing miRNAs.

**Figure 4:**
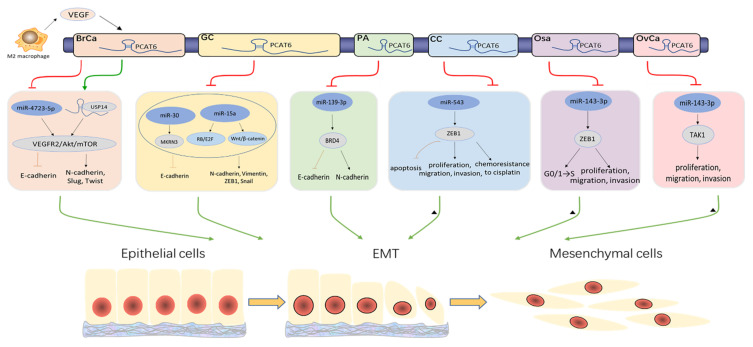
*PCAT6* expedites the EMT process. By sponging miRNAs, *PCAT6* promotes the EMT process in multiple cancers, including BrCa, GC, PA, CC, Osa, OvCa. ▲: *PCAT6* is likely to promote the EMT process by absorbing miRNAs.

LncRNAs can participate in the process of tumor progression via various molecular mechanisms, including by interacting with DNA, RNA, and protein. They play different regulatory roles by serving as different functional molecules, such as signals, decoys, guides, and scaffolds in multiple tumor cellular processes [22,72]. In recent studies, mounting evidence indicates that the lncRNA-miRNA-mRNA axis is present in cancers [73,74,75,76,77]. LncRNAs can mediate competitive mRNAs crosstalk by sharing microRNA response elements (MREs), which are also known as competing endogenous RNA (ceRNA) regulatory network mechanisms. Wang et al. first confirmed the existence of the lncRNA-related ceRNA mechanism in liver cancer. They determined that lncRNA *HULC* promoted liver cancer progression by regulating the miR-372/PRKACB axis [78]. Later on, Selmena et al. proposed the ceRNA hypothesis based on previous investigations [79]. Subsequently, more and more cancer-associated lncRNAs mediated ceRNA networks have been revealed. So far, more than 11,700,000 pairs of ceRNA have been included in the starBase database (http://starbase.sysu.edu.cn/index.php (accessed on 28 July 2021)). The ceRNA regulatory networks have been proven to be the indispensable regulators of multiple tumors growth [80], and the *PCAT6*-centric ceRNA networks have attracted wide attention. *PCAT6* promotes tumorigenesis through the PCAT6/miRNA/mRNA axis, which in turn affects different biological behaviors of tumor cells [27,44]. As shown in Figure 2, Figure 3 and Figure 4, 11 miRNAs have been verified to interact with *PCAT6*, thus participating in the ceRNA networks of *PCAT6*. Meanwhile, as members of ceRNA, the miRNAs downatream of lncRNAs can directly or indirectly regulate mRNAs and thus affect the biological behaviors of tumor cells, such as proliferation, migration, invasion, apoptosis, cell cycles [27,37,40,43,44,50,53], epithelial-mesenchymal transitions (EMT) [26,33,34,50], radiosensitivity [27], and chemoresistance [29,31].

### 4.1. PCAT6 Boosts the PI3K/Akt/mTOR Signaling Pathway

Phosphatidylinositol 3-kinase (PI3K), protein kinase B (PKB, also known as Akt), the mammalian target of the rapamycin (mTOR) signaling pathway, is vital for the regulation of cell biological functions in cancers, including cell proliferation, metastasis, cell apoptosis, autophagy, and glucose and lipid metabolism [81,82,83]. They are considered potential therapeutic targets and are involved in various biological processes in neoplasms. As shown in Figure 2, Dong et al. revealed that M2 macrophages secreted the vascular endothelial growth factor (VEGF) and upregulated the *PCAT6* expression level in BrCa. *PCAT6* overexpression further induced the Akt/mTOR pathway by absorbing miR-4723-5p to elevate vascular endothelial growth factor receptor 2 (VEGFR2) levels, which in turn promoted triple-negative breast cancer (TNBC) cell proliferation, migration, invasion, and angiogenesis in vitro, as well as tumor growth, metastasis, and angiogenesis in vivo [26].Shi et al., also proved that *PCAT6* increased the expression of tumor protein D52 (TPD52) by interacting with miR-185-5p in TNBC [27]. Another study suggested that TPD52 may exert a biological function via the PI3K/Akt signaling pathway [84]. In CRC, *PCAT6* can increase the expression level of HMGA2 via absorbing miR-204, while depletion of *PCAT6* can dramatically reduce the protein levels of HMGA2, p-PI3K, and p-Akt. Inhibition of miR-204 partially abolishes the suppressive effects of *PCAT6* knockdown on these proteins. This evidence indicates that *PCAT6* activates PI3K/Akt signaling by regulating the miR-204/HMGA2 axis and further promotes proliferation of CRC cells [31]. In addition, Liu et al. confirmed that *PCAT6* was transcriptionally upregulated by the Yin Yang 1 (YY1) protein in GBM, which further activated Akt signaling by increasing insulin like growth factor 2 mRNA binding protein 1 (IGF2BP1) expression via inhibition of miR-513. Simultaneously, the stability of *PCAT6* is enhanced by interacting with IGF2BP1. Furthermore, *PCAT6*, transcriptionally activated by YY1, promoted the proliferation and prevented the apoptosis of GBM cells through the *PCAT6*/miR-513/IGF2BP1 positive loop [35]. *PCAT6* promotes HCC cell proliferation and invasion in vitro, and tumor growth in vivo by attenuating miR-326 and upregulating heterogeneous nuclear ribonucleoprotein A2/B1 (HNRNPA2B1) [38]. Moreover, enzalutamide induced *PCAT6* also promotes PCa cell proliferation, metastasis, and neuroendocrine differentiation (NED) via the miR-326/HNRNPA2B1 axis in vitro or in vivo [52]. Several studies have clarified that HNRNPA2B1 can activate the PI3K/Akt/mTOR pathway [85,86,87,88], which further indicates that *PCAT6* may induce this pathway through the ceRNA mechanism. In Osa, *PCAT6* upregulates the transforming growth factor βreceptor 1/2 (TGFBR1/2) expression by attenuating miR-185-5p, which further activates transforming growth factor β (TGF-β) pathway-related proteins such as p-SMAD, thereby promoting the proliferation, migration, and invasion of Osa cells [45]. TGF-β is verified to be associated with the PI3K/Akt signaling pathway [89,90,91]. Zhao et al., validated that *PCAT6* promoted the progression of PA through miR-139-3p/bromodomain-containing protein 4 (BRD4) axis. Interference of *PCAT6* or the accumulation of miR-139-3p impedes tumor growth, induces apoptosis and promotes the EMT process in vivo. The MiR-139-3p inhibitor significantly attenuates the regulatory effects of *PCAT6* knockdown on cell viability, proliferation, apoptosis, and cell cycle arrest. The depletion of BRD4 reverses the promoting effects of the miR-139-3p inhibitor on PA cell viability, proliferation, migration, and invasion [50]. BRD4 has been experimentally confirmed to be associated with PI3K/Akt signaling [92,93,94]. Additionally, a study has elucidated that PI3K/Akt pathway can be activated by circ_0007841/miR-338-3p/BRD4 axis [95], which indicates *PCAT6* may activate the PI3K/Akt pathway through the BRD4 ceRNA mechanism. Wang et al. clarified that *PCAT6* acted as a sponge for miR-185-5p to increase chromobox 2 (CBX2) expression and promote PDAC cell proliferation, migration, and invasion in PADC [51]. Nevertheless, CBX2 exerts biological functions by activating the PI3K/Akt signaling pathway in tumorigenesis [96]. Taken together, *PCAT6* boosts the PI3K/Akt/mTOR signaling pathway.

### 4.2. PCAT6 Promotes the Wnt/β-Catenin Signaling Pathway

The Wnt signaling pathway plays an indispensable role in embryonic development and stem cell regulation. Therefore, dysregulated Wnt signaling activity leads to a variety of serious diseases, including cancers [97,98]. Deregulation of Wnt signaling is correlated with tumor growth, metastasis, and chemoresistance, ultimately resulting in poor prognosis of patients [99,100]. As shown in Figure 3, TPD52, HNRNPA2B1, BRD4, and CBX2, which may participate in the PI3K/Akt/mTOR pathway by the ceRNA mechanism, have also been found to be related to Wnt signaling [101,102,103,104,105,106,107,108]. *PCAT6* activates Wnt/β-catenin signaling and promotes GIST cell proliferation, maintaining stemness, and hampering GIST cell apoptosis. Meanwhile, *PCAT6* upregulates the expression level of peroxiredoxin (PRDX5) by acting as a ceRNA for miR-143-3p, while downregulation of *PCAT6* will hinder GIST cell proliferation and stemness and promote cell apoptosis. The inhibition miR-143-3p or the accumulation of PRDX5 counteract the suppressive effects of loss of *PCAT6*, indicating that *PCAT6* mediates GIST cell proliferation, stemness, and apoptosis through the miR-143-3p/PRDX5 axis [32]. Additionally, it has been revealed that PRDX5 influences cellular biological behavior by activating the Wnt/β-catenin pathway [109], which suggests that *PCAT6* can activate the Wnt/β-catenin pathway through. the ceRNA network of PRDX5. Dong et al. discovered that *PCAT6* excited Wnt/β-catenin and RB/E2F signaling by targeting miR-15a in GC. Decreased expression of *PCAT6* represses GC cell proliferation and accelerates cell apoptosis, whereas a miR-15a inhibitor reverses the performance [33]. Su et al. found that sevoflurane inhibited LC cell proliferation, migration, and invasion, conversely accelerating cell apoptosis, and inactivated the Wnt/β-catenin pathway. However, *PCAT6* abolishes the effects of sevoflurane on cell biology function. Analogously, miR-326, acting as a ceRNA for *PCAT6*, also rescues the effects of *PCAT6* on sevoflurane-treated cells. Furthermore, Wnt5a, a downstream target of miR-326, restores the regulatory role of miR-326 in LC cells. Hence, sevoflurane inhibits the *PCAT6* that absorbs miR-326, further downregulates Wnt5a, deactivates Wnt/β-catenin signaling, and impairs LC cell biology function [42].

### 4.3. PCAT6 Facilitates the EMT Process

The epithelial-to-mesenchymal transition (EMT), an indispensable cellular program, exerts a specific role in embryonic development and tissue homeostasis. The EMT process is activated by wound healing, fibrosis, and tumors. The EMT process, which is aberrantly induced by cancer cells, enhances cell metastatic and invasive capacity [110,111,112,113]. Acting as a sponge for miR-4723-5p in TNBC, *PCAT6* upregulates the VEGFR2/Akt/mTOR signaling pathway, leading to significantly decreased E-cadherin expression and significantly increased N-cadherin, Slug, and Twist expression (Figure 4). Hence, *PCAT6* enhances cell migration and invasion by partly accelerating the transformation of the EMT process [26]. It has been revealed that *PCAT6* upregulates makorin ring finger protein 3 (MKRN3) by endogenously competing with miR-30 in GC. The introduction of *PCAT6* impairs the protein expression of caspase-3, caspase-9, Bax, and E-cadherin, and promotes the expression of Bcl-2, N-cadherin, Vimentin, zinc finger E-box binding homeobox 1 (ZEB1), and Snail, which validates the indication that *PCAT6* facilitates the GC cell EMT process and hampers apoptosis via the miR-30/MKRN3 axis [34]. Similarly, Dong et al. also found that *PCAT6* decreased GC cell apoptosis and EMT by endogenously competing with miR-15a. Deficiency of *PCAT6* promotes apoptosis by conspicuously restraining the Cyclin D1 protein level and enhancing the protein levels of p53, Bax, and cleaved caspase-3. The silence of *PCAT6* impedes cell EMT process by decreasing the expression of N-cadherin, Vimentin, Snail, and ZEB1 except for E-cadherin [33]. This evidence proves that *PCAT6* induces the EMT process by the ceRNA mechanism in GC. Moreover, the results of a subcutaneous xenotransplanted PA mice model have demonstrated that *PCAT6* knockdown and miR-139-3p overexpression can induce the increase of the E-cadherin protein level and the decrease of the N-cadherin level in tumor tissues. The inhibition of miR-139-3p reverses its suppressive effect and decrease of *PCAT6* on the PA cell EMT process in vitro [50]. ZEB1, as a transcriptional regulator, has been confirmed to be involved in the EMT process [114]. It also have observed that lncRNAs affect the EMT process by regulating the miRNAs/ZEB1 axis [115,116]. In CC, *PCAT6* promotes cell proliferation and metastasis in vitro, and tumor growth in vivo through the miR-543/ZEB1 axis [29]. In addition, *PCAT6* also upregulates the ZEB1 level by absorbing miR-143-3p in Osa, promoting cell proliferation, migration, invasion, and cell cycle in vitro, and tumor growth in vivo [44]. Hence, *PCAT6* participates in the EMT process by regulating the miRNAs/ZEB1 axis and further influences cell migration and invasion. Moreover, *PCAT6* also targets miR-143-3p to activate TGF-β-activated kinase 1 (TAK1) in OvCa, and then accelerates cell proliferation and enhances cell metastasis [48]. Previously, TAK1 has been reported to induce the EMT process in various diseases [117,118]. Therefore, these results confirm that *PCAT6* regulates the EMT process by acting as a ceRNA for miRNAs. 

### 4.4. PCAT6 Leads to Radioresistance and Chemoresistance

Currently, radiotherapy remains the preferred therapy for many cancers. However, many patients who are resistant to radiation have poor prognosis, with cancer recurrence and attenuating radiotherapy effectiveness [119]. Hence, the radiosensitivity of patients is the key to radiotherapy for malignant tumors. Radioresistance is a complex process involving various molecular mechanisms [120]. Recently, increasing amounts of evidence have demonstrated that many lncRNAs are closely associated with radiotherapies, including *RBM5-AS1* [121], *HOTAIR* [122], and *PVT1* [123]. Shi et al. also found that there was a relationship between *PCAT6* and radioresistance in TNBC. The interference of *PCAT6*, up-regulation of miR-185-5p, or inhibition of TPD52 enhances the radiosensitivity of TNBC cells, inhibits cell proliferation, arrests the cell cycle in the G_0_/G_1_ phase, and accelerates cell apoptosis (Figure 2) [27]. 

Chemotherapy is an effective anticancer treatment. However, patients can also develop resistance to drug treatment like radioresistance, leading to a dismal prognosis [124,125]. Chemoresistance is influenced by tumor properties, biological behavior, and microenvironments [126]. To date, many drugs have been used in chemotherapy, such as platinum, paclitaxel, and 5-fluorouracil (5-FU). Exploring new mechanisms of chemotherapeutic drugs to overcome chemoresistance is urgently needed. Recently, lncRNA dysregulation has been associated with drug resistance, including *UCA1* [127], *CYTOR* [128], and *TINCR* [129]. Furthermore, the aberrant expression of *PCAT6* was also verified to be related to chemoresistance in cancers. It has been demonstrated that both the intervention of *PCAT6* and the introduction of miR-543 impair CC cell chemoresistance to cisplatin. Moreover, *PCAT6* rescues the suppressive effect of miR-543 on cell chemoresistance [29]. ZEB1, with significant negative correlation with miR-543 and with positive correlation with *PCAT6*, was confirmed to be a downstream target of miR-543 [29]. Furthermore, the overexpression of ZEB1 partially counteracts the inhibitory effect of *PCAT6* downregulation on cell chemoresistance to cisplatin (Figure 4) [29]. Wu et al. found that loss of *PCAT6* reversed the chemoresistance of CRC cells to 5-fluorouracil (5-FU), while this performance could be partially abrogated by depression of miR-204. Moreover, *PCAT6* activates HMGA2/PI3K signaling by absorbing miR-204, thus reducing the CRC cells’ chemosensitivity to 5-FU (Figure 2) [31]. Consequently, *PCAT6* may be a new effective target for treatment in cancer patients undergoing clinical radiotherapy and chemotherapy resistance.

### 4.5. Other ceRNA Mechanisms of PCAT6

In addition to those mentioned above, it has been elucited that *PCAT6* boosts cancer progress via various signal pathways. *PCAT6* can act as a sponge for miR-513a to facilitate BC progression. The inhibition of miR-513a partially offsets the proliferation, migration, and invasion induced by the knockdown of *PCAT6* [21]. Moreover, *PCAT6* promotes cell proliferation, migration, and invasion by inhibiting miR-143-3p and upregulating protein disulfide isomerase familyA mumber 6 (PDIA6), in BC [25]. In NSCLC *PCAT6* absorbs miR-330-5p and promotes cell proliferation, migration, invasion in vitro, and tumor growth in vivo [39]. *PCAT6* has also been found to have a competitive binding relationship with miR-330-5p, which in turn induced CCA cell proliferation and invasion [49]. 

**Figure 5:**
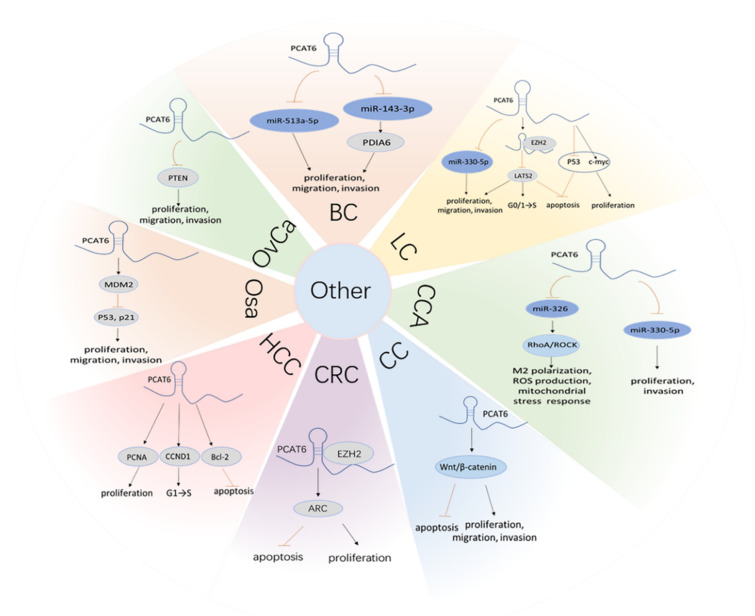
*PCAT6* exerts biological behaviors by other ceRNA and beyond ceRNA mechanisms in various cancers, including BC, LC, CCA, CC, CRC, HCC, Osa, OvCa.

Furthermore, increasing evidence has indicated that dozens of lncRNAs are associated with immune cell function. For instance, *XIST* [130] and *SBF2-AS1* [131] can the induce M2 polarization of tumor-associated macrophages, which are indispensable cells within the tumor immune microenvironment. Similarly, Tu et al. validated that *PCAT6* overexpression induced the M2 polarization of macrophages deriving from CCA patients, and indicated the effect could be reversed by miR-326. Simultaneously, miR-326 mimics can abolish the promoting impacts of *PCAT6* introduction on macrophage cellular reactive oxygen species production and mitochondrial and metabolic dysfunction. Furthermore, RhoA has been confirmed to be a downstream target of miR-326, which further activates the RhoA-ROCK signaling pathway and promotes tumor progression. The results of animal experiments uncovered that depletion of *PCAT6* inhibited tumor growth by activating the T cell response in vivo, which was mainly manifested by the increase of CD3^+^ T cells and IFN-γ-producing CD4^+^ T and CD8^+^ T cells. These findings suggest that *PCAT6* may serve as a potential immune-therapeutic target for CCA (Figure 5) [54]. 

## 5. The Mechanisms of *PCAT6* above ceRNA 

In addition to absorbing miR-4723-5p, *PCAT6* also activates the VEFGR2/Akt/mTOR axis by recruiting USP14 to stabilize VEGFR2 and further elicits biological functions in vitro and in vivo in BrCa (Figure 2) [26]. TOP/FOP flash reporter assays have revealed that silence of *PCAT6* inactivates Wnt/β-catenin signaling pathway in CC. Meanwhile, the results from real-time fluorescence quantitative PCR (qRT-PCR) and Western blot analyses indicate that several pivotal genes of Wnt/β-catenin signaling (e.g., β-catenin, cyclin D1, and c-myc), are remarkably suppressed in CC cells transfected with siRNA targeting for *PCAT6* [28]. *PCAT6* upregulates the activity regulated cytoskeleton associated protein (ARC) via binding to EZH2 in CRC. It also impedes cell apoptosis and promotes cell proliferation [30]. In HCC, *PCAT6* promotes cell proliferation, increases the proportion of the S phase, and decreases cell apoptosis. In addition, *PCAT6* also affects the expression of the protein related to the proliferation, cycle, and apoptosis, including proliferating cell nuclear antigen (PCNA), cyclin D1 (CCND1), and B-cell lymphoma-2 (Bcl-2) [37]. Bioinformatics analyses indicate that *PCAT6* may be associated with Wnt, HIF-1, and metabolic pathways. Also, it may correlate with p53 and participate in the apoptotic signaling pathways [37]. *PCAT6* indirectly regulates c-myc and p53 genes to affect Bcl-2 and Bax expressions, which further prevent sLC cell early apoptosis and induce cell proliferation [20]. Moreover, in NSCLC, *PCAT6* transcriptionally impairs large tumor suppressor kinase 2 (LATS2) promoter activity by binding to EZH2, which mediates H3K27 trimethylation and accelerates the cell cycle, promoting cell proliferation, tumor growth and metastasis, and hindering cell apoptosis [40]. In Osa, overexpression of *PCAT6* enhances cell proliferation, migration, and invasion in vitro, and tumor growth in vivo, while depression of MDM2 proto-oncogene (MDM2) exerts an opposite influence. Moreover, upregulation of *PCAT6* decreases p53 and p21 expression, and further induces tumorgenesis [46]. However, this performance is rescued by the depletion of MDM2 [46]. In OvCa, *PCAT6* increases the capacity of cell proliferation and metastasis by decreasing the phosphatase and tensin homolog (PTEN) level. Moreover, both the mRNA and protein levels of PTEN are upregulated by depleting *PCAT6*. Meanwhile, knockdown of PTEN rescues the inhibition of the metastatic capability of OvCa cells induced by *PCAT6* suppression (Figure 5) [47]. Lang et al. discovered that *PCAT6* upregulating by methyltransferase 3 (METTL3), a catalytic subunit of the N6-adenosine-methyltransferase complex that induces m^6^A modification, promoted PCa cell proliferation and metastasis in vitro, and accelerated tumor growth and bone metastasis (BM) in vivo. Upregulation of *PCAT6* can interact with insulin like growth factor 2 mRNA binding protein 2 (IGF2BP2) and insulin like growth factor 1 receptor (IGF1R) to stabilize IGF1R mRNA and activate the downstream signaling pathways, including PI3K/Akt signaling and NF-κB signaling, which ultimately facilitate tumor growth and BM (Figure 2) [53].

## 6. The Diagnostic and Prognostic Value of *PCAT6* Overexpression

Various studies have shown that high expression of *PCAT6* in multiple cancers is associated with various clinicopathologic features, mainly including larger tumor size, high tumor differentiation, advanced tumor stage, more tissues metastasis, and so on (Table 2)., Some researchers have estimated the diagnostic and prognostic values of upregulated *PCAT6* in cancers (Table 3). Zhang et al. found that the area under the curve (AUC) of the serum *PCAT6* level was greater than 0.8, which could distinguish BC patients from the normal controls. It has been further demonstrated that the serum *PCAT6* level is suitable as a BC diagnosis biomarker [24]. Wan et al. revealed that the tissue *PCAT6* level had great diagnostic values, including AUC greater than 0.9, with higher sensitivity (86.67–100%) and higher specificity (78.57–96%) in 349 cases of NSCLC from five GEO datasets (GSE19804, GSE18842, GSE30219, GSE19188, and GSE27262). Meanwhile, they also had noticed that the AUCs of plasma *PCAT6* were 0.9213 (sensitivity, 87.67%; specificity, 97.44%) in lung adenocarcinoma (LUAD) and 0.9583 (sensitivity, 94.12%; specificity, 100%) in lung squamous cell carcinoma (LUSC), respectively [41]. These results suggest that *PCAT6* may be a promising diagnostic biomarker for BC and LC.

In addition, more studies have found that the accumulation of *PCAT6* is closely related to the prognosis of various tumor patients. It has been illustrated that the *PCAT6* level is negatively correlated to the ovarall survival (OS) of patients with CRC [30], GC [34], LC [20,40,43], and PDAC [51]. Furthermore, increased expression of *PCAT6* reduces OS and progression-free survival (PFS) in BC [21,24] and Osa [44,45] patients. Lv et al. conducted Kaplan–Meier survival analysis to investigate the association between *PCAT6* level and postoperative survival in patients with CC. They found that a high level of *PCAT6* predicts poor OS and disease-free survival (DFS) in patients. Subsequently, univariate analysis and multivariate analysis also have revealed that high expression of *PCAT6* is an independent poor prognostic factor for both OS and DFS in CC [28]. For HCC, many reports have revealed that upregulated *PCAT6* is associated with shorter OS, PFS, and DFS [36,37,38,132]. Similarly, Kaplan–Meier Plotter analyses have demonstrated that high expression of *PCAT6* can be used as a prognostic biomarker for OS, PFS, and post-progression survival (PPS) in OvCa patients [48]. In PCa patients with BM, patients with higher *PCAT6* expression have poorer overall and BM-free survivals and shorter DFS [53]. These results have indicated that *PCAT6* may be a clinical prognostic factor. 

## 7. Discussion and Perspectives

In conclusion, LncRNA *PCAT6*, a vital oncogene, is first found overexpressed in lung cancer [20], and subsequently verified in multiple human cancers. It has been proven to be a promising diagnostic biomarker and clinical prognostic factor in different diseases (Table 3). The accumulation of *PCAT6* is closely related to many clinicopathological features (Table 2). Furthermore, *PCAT6* overexpression promotes tumor cell proliferation [21], migration [44], invasion [45], chemoresistance [29,31], and radioresistance [27]. These processes involve various signaling pathways, including PI3K/Akt/mTOR signaling, Wnt/β-catenin signaling, signaling, and RhoA-ROCK signaling. It has been confirmed that these pathways play indispensable roles in tumorigenesis and disease progression [133,134,135,136]. However, *PCAT6* can regulate the signaling pathways through ceRNA [26] and other mechanisms [28]. Hence, *PCAT6*, as a promising diagnostic biomarker, may be a checkpoint target for precision therapy in human cancers. 

## Figures and Tables

**Table 1:** Functional characterization of *PCAT6* in multiple human cancers.

Tumor Types	Expression	Sample Type	Role	Functional Role in Vitro	Functional Role in Vivo	Related Genes/Protein/Pathways	Ref.
Bladder cancer	Up	cells (RT4, T24, J82, UMUC3, 5637), patient tissue and serum	Tumor promoter	Cell proliferation and apoptosis		NA	[24]
	Up	Cells (T24, EJ, 253j, 5637), patient tissue	Tumor promoter	Cell proliferation, migration, and invasion		miR-513a-5p	[21]
	Up	Cells (T24T, EJ, UMUC3, 5637), patient tissue	Tumor promoter	Cell proliferation, migration, and invasion		miR-143-3p, PDIA6	[25]
Breast cancer	Up	Cells (MDA-MB-231, MDA-MB-468, MDA-MB-436, HCC-1937), patient tissue	Tumor promoter	Cell proliferation, migration, invasion, EMT process, and angiogenesis	Tumor growth, metastasis, and angiogenesis	VEGF, VEGFR2/Akt/mTOR, miR-4723-5p, USP14, E-cadherin, N-cadherin, Slug, Twist,	[26]
	Up	Cells (MDA-MB-468, MDA-MB-231), patient tissue	Tumor promoter	Cell proliferation, apoptosis, cell cycle, and radiosensitivity		miR-185-5p, TPD52	[27]
Cervical cancer	Up	Cells (Caski, SW756, HeLa, ME-180, SiHa, C33A), patient tissue	Tumor promoter	Cell proliferation, apoptosis, migration, and invasion		Wnt/β-catenin, β-catenin, cyclin D1, c-myc	[28]
	Up	Cells (SiHa, HeLa, ME180, C-33A), patient tissue	Tumor promoter	Cell proliferation, apoptosis, migration, invasion, and chemoresistance	Tumor growth	miR-543, Bcl-2, cleaved-caspase 3, ZEB1	[29]
Colorectal cancer	Up	Cells (SW628, SW480, RKO, COLO320HSR, HCT116), patient tissue	Tumor promoter	Cell proliferation and apoptosis	Tumor growth	Cleaved-caspase 3, ARC, EZH2	[30]
	Up	Cells (HCT116, HT-29, SW620, SW480, DLD-1, RKO, LoVo), patient tissue	Tumor promoter	Cell proliferation and chemoresistance		miR-204, HMGA2, PI3K, Akt	[31]
Gastrointestinal stromal tumor	Up	Cells (GIST-H1, GIST-882, GIST-T1, GIST-48), patient tissue	Tumor promoter	Cell proliferation, stemness, and apoptosis		Wnt/β-catenin, miR-143-3p, PRDX5	[32]
Gastric cancer	Up	Cells (BGC-823, SGC-7901, HGC-27, MKN45), patient tissue	Tumor promoter	Cell proliferation, migration, EMT, and apoptosis		miR-30, MKRN3, caspase 3, caspase 9, Bax, Bcl-2, E-cadherin, N-cadherin, Vimentin, ZEB1, Snail	[34]
	Up	Cells (MKN45, SGC-7901, AGS, MKN28), patient tissue	Tumor promoter	Cell proliferation, EMT, and apoptosis		Cyclin D1, p53, Bax, cleaved caspase 3, E-cadherin, N-cadherin, Vimentin, Snail, ZEB1, miR-15a, RB/E2F, Wnt/β-catenin	[33]
Glioblastoma	Up	Cells (A172, U251, U87, LN229), patient tissue	Tumor promoter	Cell proliferation and apoptosis		YY1, miR-513, IGF2BP1, Akt	[35]
Hepatocellular carcinoma	Up	Patient tissue	Tumor promoter	Cell proliferation and migration		NA	[36]
	Up	Cells (HuH7, SMMC-7721, Hep3B, HepG2, PLC/PRF/5), patient tissue	Tumor promoter	Cell proliferation, cycle, apoptosis, and migration		PCNA, CCND1, Bcl-2	[37]
	Up	Cells (MHCC97H, HepG2, Huh7), patient tissue	Tumor promoter	Cell proliferation and invasion	Tumor growth	miR-326, hnRNPA2B1	[38]
Lung cancer	Up	Cells (H1650, HCC827, H1975, A549), patient tissue	Tumor promoter	Cell proliferation, migration, and invasion,	Tumor growth	miR-330-5p	[39]
	Up	Cells (SK-MES-1, H1703, H520, H1299, H1975, SPCA1, A549), patient tissue	Tumor promoter	Cell proliferation, cycle, apoptosis, migration, and invasion	Tumor growth	EZH2, LATS2	[40]
	Up	Cells (H292, PC-9, CL1-5, H460, H1650, A549, H446, H1975)	Tumor promoter	Cell proliferation, apoptosis, and invasion		Bcl-2, Bax, c-myc, p53	[20]
	Up	Cells (H446, H1975), patient tissue	Tumor promoter	Cell proliferation, migration, invasion, and apoptosis		c-myc, MMP9, cleaved-caspase-3, Wnt5a, β-catenin. miR-326.	[42]
	Up	Cells (H1838, H522, H2228, H358, H1299, A549),	Tumor promoter	Cell proliferation, migration, invasion, cycle, apoptosis	Tumor growth	Caspase-3, Ki-67	[43]
Osteosarcoma	Up	Cells (MG-63, Saos-2, 143B, U2OS), patient tissue	Tumor promoter	Cell proliferation, migration, invasion, and cell cycle	Tumor growth	ZEB1, miR-143-3p	[44]
	Up	Cells (Saos2, MG63, U2OS, HOS)	Tumor promoter	Cell proliferation, migration, and invasion	Tumor growth	MMP2, MMP9, p53, p21, MDM2	[46]
	Up	Cells (Saos2, HOS, U2OS, 143B, KHOS/240S, MG63, SK-ES-1), patient tissue	Tumor promoter	Cell proliferation, migration, and invasion		miR-185-5p, TGF-β, p-SMAD, TGFBR1/2	[45]
Ovarian cancer	Up	Cells (OVCAR3, PEO1, A2780, 3AO, CAOV3, SKOV3), patient tissue	Tumor promoter	Cell proliferation, migration, and invasion		PTEN	[47]
	Up	Patient tissue	Tumor promoter	Cell proliferation, migration, and invasion		miR-143-3p, TAK1	[48]
Cholangiocarcinoma	Up	Patient-derived macrophages, patient tissue	Tumor promoter	M2 polarization of macrophages, cellular reactive oxygen species production, mitochondrial and metabolic dysfunction	Tumor growth	miR-326, RhoA, ROCK1, ROCK2	[54]
	Up	Cell (ICC-9810, CCLP1, HuCC-T1, QBC939), patient tissue	Tumor promoter	Cell proliferation and invasion		miR-330-5p	[49]
Pituitary adenomas	Up	Patient tissue	Tumor promoter	Cell proliferation, migration, invasion, viability, apoptosis, cell cycle, and EMT	Tumor growth, apoptosis, EMT	miR-139-3p, BRD4, E-cadherin, N-cadherin, Bcl-2, Bax, cleaved-caspase 3	[50]
Pancreatic ductal adenocarcinoma	Up	Cell (Capan-2, AsPC-1, PANC1, BxPC-3), Patient tissue	Tumor promoter	Cell proliferation, migration, and invasion		miR-185-5p, CBX2	[51]
Prostate cancer	Up	Cell (NCI-H660), patient tissue	Tumor promoter	Cell NED, proliferation, and invasion	Tumor growth and metastasis	NSE, SYP, ChgA, miR-326, hnRNPA2B1	[52]
	Up	Patient tissue	Tumor promoter	Cell proliferation, cycle, migration, and invasion	Tumor growth and BM	IGF2BP2, IGF1R, PI3K/Akt, NF-κB, METTL3, ALKBH5	[53]

PDIA6, protein disulfide isomerase family A number 6; EMT, epithelial-mesenchymal transition; VEGF, vascular endothelial growth factor; VEFGR2, vascular endothelial growth factor receptor 2; Akt, serine/threonine kinase; mTOR, mammalian target of rapamycin; USP14, ubiquitin-specific protease-14; TPD52, tumor protein D52; Bcl-2, B-cell lymphoma-2; ZEB1, zinc finger E-box binding homeobox 1; ARC, activity regulated cytoskeleton associated protein; EZH2, enhancer of zeste homolog 2; HMGA2, high mobility group AT-hook 2; PRDX5, peroxiredoxin 5; MKRN3, makorin ring finger protein 3; YY1, Yin Yang 1; IGF2BP1, insulin like growth factor 2 mRNA binding protein 1; IGF2BP2, insulin like growth factor 2 mRNA binding protein 2; PCNA, proliferating cell nuclear antigen; CCND1, cyclin D1; hnRNPA2B1, heterogeneous nuclear ribonucleoprotein A2/B1, LATS2, large tumor suppressor kinase 2; Bax, BCL2 associated X; MMP2, matrix metallopeptidase 2; MMP9, matrix metallopeptidase 9; MDM2, mouse double minute 2 homolog; TGF-β, transforming growth factor β; TGFBR1/2, transforming growth factor β receptor 1/2; PTEN, phosphatase and tensin homolog; TAK1, TGF-β activated kinase 1; RhoA, ras homolog family member A; ROCK1/2, Rho associated coiled-coil containing protein kinase 1/2; BRD4, bromodomain containing 4; CBX2, chromobox 2; NED, neuroendocrine differentiation; SYP, synaptophysin; ChgA, chromogranin A; BM, bone metastasis; IGF1R, insulin like growth factor 1 receptor; METTL3, methyltransferase 3, N6-adenosine-methyltransferase complex catalytic subunit; ALKBH5, alkB homolog 5.

**Table 2:** Clinicopathologic features of *PCAT6* in multiple human cancers.

Tumor Types	Clinicopathologic Features	Ref.
Bladder cancer	Larger tumor size, high tumor differentiation, advanced TNM stage, more lymph nodes metastasis, more distant metastasis	[24]
	Pathological stage	[21]
Breast cancer	More tissues metastasis, higher tumor stages	[26]
	More lymph nodes metastasis, advanced tumor stages	[27]
Cervical cancer	Advanced FIGO stage, more lymph nodes metastasis, depth of cervical invasion	[28]
Colorectal cancer	Tumor subtype, N classification, metastasis, poorer clinical stage	[30]
	Larger tumor size, advanced TNM stage, lymph node metastasis	[31]
Gastric cancer	Larger tumor size, advanced TNM stage, more metastasis	[34]
Hepatocellular carcinoma	Moderated or poorly differentiation, advanced TNM stage	[37]
Lung cancer	Advanced TNM stage, more metastasis	[41]
	Advanced TNM stage, more metastasis	[39]
	Larger tumor size, more lymph node metastasis, advanced TNM stage	[40]
	Larger tumor size, more lymph node metastasis, advanced TNM stage	[20]
	Larger tumor size, more metastasis, advanced TNM stage	[43]
Osteosarcoma	Larger tumor size, more metastasis, advanced TNM stage	[44]
	Larger tumor size, more metastasis, advanced TNM stage	[45]
Ovarian cancer	More lymph node metastasis, more distant metastasis	[47]
	Advanced TNM stage	[48]
Cholangiocarcinoma	Advanced TNM stage	[49]
Pancreatic ductal adenocarcinoma	Advanced TNM stage, more lymph node invasion	[51]
Prostate cancer	Poor differentiation, higher serum PSA, advanced gleason grade, more BM	[53]

TNM, tumor node metastasis; FIGO, Federation of Gynecology and Obstetrics; PSA, prostate specific antigen; BM, bone metastasis.

**Table 3:** Diagnostic or prognostic value of *PCAT6*.

Tumor Types	Expression	Diagnostic or Prognostic Value	Ref.
Bladder cancer	Up	Poor OS, and poor PFS, AUC > 0.8	[24]
	Up	Poor OS	[21]
Cervical cancer	Up	Shorter OS and DFS	[28]
Colorectal cancer	Up	Worse OS	[30]
	Up	Poor OS	[31]
Gastric cancer	Up	Worse prognosis	[34]
Hepatocellular carcinoma	Up	Poor OS	[36]
	Up	Poor OS and DFS	[37]
	Up	Shorter OS	[38]
	Up	Poor PFS	[132]
Lung cancer	Up	Tissue *PCAT6*: AUC > 0.9, sensitivity 86.67–100%, specificity 78.57–96%Plasma *PCAT6*: a. LUAD: AUC = 0.9213, sensitivity 87.67%, specificity 97.44; b. LUSC: AUC = 0.9583, sensitivity 94.12%, specificity 100%	[41]
	Up	Poor OS	[40]
	Up	Poor OS	[20]
	Up	Poor OS	[43]
Osteosarcoma	Up	Shorter OS and PFS	[44]
	Up	Shorter OS and PFS	[45]
Ovarian cancer	Up	Poor OS, PFS and PPS	[48]
Pancreatic ductal adenocarcinoma	Up	Worse OS	[51]
Prostate cancer	Up	Shorter overall and BM-free survival, shorter DFS	[53]

OS, overall survival; PFS, progression-free survival; AUC, area under curve; DFS, disease-free survival; PPS, post progression survival.
